# Anticancer Effect of STING Agonist-Encapsulated Liposomes on Breast Cancer

**DOI:** 10.3390/molecules28093740

**Published:** 2023-04-26

**Authors:** Jibing Zhang, Xiao Cui, Yujiao Huang, Xiangdong Xu, Changshun Feng, Jun Li

**Affiliations:** 1 School of Pharmaceutical Sciences, Liaocheng University, Liaocheng 252059, China; zjbdare@163.com (J.Z.); 15562897872@163.com (X.C.); fcs991120@163.com (C.F.); 2 School of Life Sciences, Beijing University of Chinese Medicine, Beijing 102488, China; yujiao_huang@163.com; 3 Liaocheng Inspection and Testing Center, Liaocheng 252000, China; xxd0817@126.com

**Keywords:** STING, agonist, liposomes, antitumor, breast cancer

## Abstract

Breast cancer is one of the most common cancers worldwide, posing a serious threat to human health. Recently, innate immunity has become a widely discussed topic in antitumor research. The STING pathway is an important component of innate immunity, and several STING agonists have been developed and applied in antitumor research. Dimeric amidobenzimidazole (diABZI) is one STING agonist and is a nucleotide analog with low serological stability and cell membrane permeability. In this study, we prepared diABZI-encapsulated liposomes (dLNPs) using the ammonium sulfate gradient method. The average particle size of the dLNPs was 99.76 ± 0.230 nm, and the encapsulation efficiency was 58.29 ± 0.53%. Additionally, in vivo and in vitro assays showed that the dLNPs had a sustained-release effect and that the circulation time in vivo was longer than 48 h. The expression of IFN-β and IFN-γ was elevated in mice treated with dLNPs. Moreover, we found that dLNPs can recruit CD8^+^ T cells to tumor tissue and exert antitumor effects. The dLNPs-treated group showed the most significant efficacy: the average tumor volume was 231.46 mm^3^, which decreased by 78.16% and 54.47% compared to the PBS group and diABZI group. Meanwhile, the hemolysis rate of the dLNPs was 2%, showing high biocompatibility. In conclusion, dLNPs can effectively suppress tumor growth and possess great potential in breast cancer therapy.

## 1. Introduction

Breast cancer has become the most commonly diagnosed cancer in the world, posing a serious threat to women’s health and lives [1]. Until now, chemotherapy and surgical resection have remained the standard treatments for breast cancer [2]. However, breast cancer is prone to recurrence or metastasis after chemotherapy and surgical removal. Therefore, it is necessary to develop other safe and effective treatment methods.

In the last 30 years, research on cancer immunotherapy has been in full swing and has achieved great success in clinical settings. Cancer immunotherapy includes both immune normalization and immune enhancement. Immune enhancement mediated by the activation of innate immunity has become a hot topic in antitumor research. The stimulator of interferon genes (STING) plays an important role in innate immunity [3,4,5]. The STING pathway can be activated by binding agonists to the STING protein located on the endoplasmic reticulum membrane [6,7]. After the STING activation, TBK1/IRF3 signal-dependent type Ⅰ IFN is released. Meanwhile, CD8^+^ T cell activation is induced by the type I interferon. Subsequently, IFN-γ can be expressed by the CD8^+^ T cells. The abovementioned interferons induce the remodeling of the tumor immune microenvironment [8,9].

STING agonists, a class of cyclic dinucleotide (CDN) analogs, were developed to trigger STING pathway activation [10]. In addition, STING agonists are also potential therapeutic cancer vaccine adjuvants [11,12]. Dimeric amidobenzimidazole (diABZI) is a novel STING agonist that can be administered intravenously. Regrettably, diABZI has low serological stability, a net negative charge, and poor cell membrane penetration [13]. As a result, the low concentrations of diABZI that are distributed in the cytoplasm cannot effectively activate STING molecules on the endoplasmic reticulum membrane [14,15]. Therefore, changing dosage forms is urgently needed to improve the antitumor efficiency of diABZI. The development of nanoliposome formulation technology has made it possible to overcome these deficiencies.

Nanoliposome formulation technology has become an essential approach for the targeted, long-lasting, and combined delivery of antitumor drugs. It has the advantages of high drug encapsulation efficiency and excellent stability in vitro and in vivo [16,17,18]. Liposomes are mainly composed of phospholipids, which are the main components of biological membranes and have high biocompatibility. At the same time, liposomes protect the drug from being broken down by enzymes in the body [19]. Liposomes are widely used for the delivery of antitumor drugs and have achieved great therapeutic efficacy in tumor models such as prostate cancer and melanoma [11,12,20,21,22]. Thus, nanoliposome formulation technology is a potential strategy to improve the antitumor effects of diABZI.

In this study, diABZI-encapsulated liposomes (dLNPs) with a high encapsulation efficiency and drug-loading capacity were prepared using the ammonium sulfate gradient method. dLNPs can accumulate in tumor tissue owing to their enhanced permeability and retention effect (EPR) effect [23]. Passive targeting can reduce tissue toxicity and increase the cellular uptake efficiency by macrophages [24]. Compared with free diABZI, the dLNPs exhibited more efficient STING activation and a stronger antitumor effect.

## 2. Results

### 2.1. Preparation and Characterization of Liposomes

**Figure 1:**
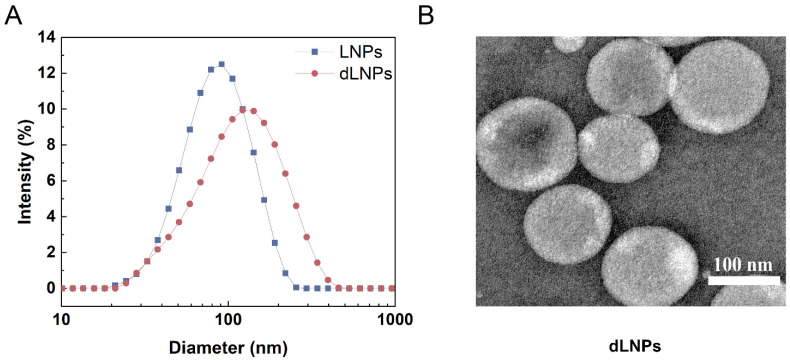
Liposome characterization. (**A**) The particle size of LNPs and dLNPs (*n* = 3). (**B**) TEM images of dLNPs. Scale bar: 100 nm.

Liposome particle size is an important indicator for evaluating liposomes, and, when the particle size is less than 150 nm, it has higher stability and longer circulation time in vivo. The average particle sizes of the LNPs and dLNPs demonstrated by the DLS were 81.14 ± 0.586 nm and 99.76 ± 0.230 nm, respectively. The polydispersity index (PDI) of the LNPs and dLNPs was 0.209 ± 0.0196 and 0.225 ± 0.0076, respectively. The zeta potential of the LNPs and dLNPs was −8.96 ± 0.466 mV and −6.74 ± 0.896 mV, respectively (Table 1). The morphology of the dLNPs was examined using TEM. As shown in Figure 1B, the diameter of the dLNPs was about 100 nm. The encapsulation efficiency of the dLNPs was 58.29 ± 0.53%, as obtained from the HPLC analysis. The drug loading of the dLNPs was 174.87 ± 1.59 mg/mL.

### 2.2. In Vitro Drug Release of dLNPs

**Figure 2:**
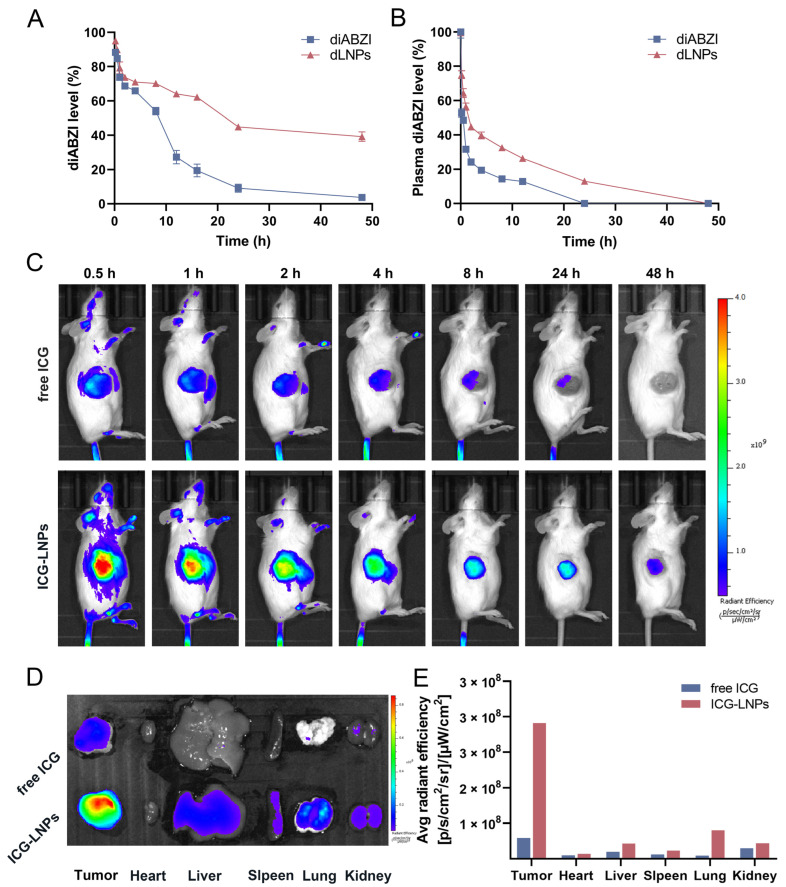
Release rate and passive targeting of dLNPs. (**A**) The release profile of diABZI and dLNPs in PBS at 37 °C). (**B**) The profile of diABZI in plasma at 10 min, 30 min, 1 h, 2 h, 4 h, 8 h, 12 h, 24 h, 36 h, and 48 h after administration. (**C**) Fluorescence imaging of ICG in mice after intravenous injection of free ICG and ICG-LNPs. (**D**) Fluorescence imaging of ICG in tumor, heart, liver, spleen, lung, and kidney after intravenous injection of free ICG and ICG-LNPs, respectively. (**E**) Average radiant efficiency of tumor and main organs corresponding to (**D**).

As shown in Figure 2A, the release rate of the diABZI was 95.07% at 48 h, while that of the dLNPs was 56.75%. This result suggests that the dLNPs had a slow-release effect.

### 2.3. Pharmacokinetic Study

Compared with the free diABZI, the dLNPs were released slowly. In general, the blood concentration of the free diABZI group was lower than that of the dLNPs group, except in the early stage after administration (Figure 2B). Additionally, the blood concentration of the diABZI group decreased rapidly from 0 to 1 h after administration, whereas the decrease was slower in the dLNPs group. Moreover, the blood concentration of the diABZI group decreased to 0 after 12 h of intravenous administration, while it was maintained at 12.9% in the dLNPs group. The dLNPs decreased the drug clearance rate and exhibited better sustained-release performance than free diABZI.

### 2.4. In Vivo Passive Targeting Efficiency of LNPs

Fluorescence images of the mice were obtained after injection with free ICG or ICG-LNPs. At 48 h, the ICG in the tumor tissue of mice in the free ICG group had been metabolized in vitro, while, in the ICG-LNPs group, the ICG in the tumor tissue was still at a high level (Figure 2C). In addition, the tumor tissue and the main organs of the mice were harvested at 48 h and were imaged in an optical imaging system for living animals. The fluorescence intensity of the organs in the free ICG group was lower than it was in the ICG-LNPs group, especially in the tumor tissues (Figure 2D). The fluorescence intensity of the tumor tissues in the ICG-LNPs group was about six times that of the free ICG group (Figure 2E), illustrating that LNPs can passively target tumor tissues and prolong circulation time in vivo. Previous studies have shown that intravenously injected liposomes are able to passively target tumor tissue due to the EPR effect.

### 2.5. The Preventive Effect of dLNPs

**Figure 3:**
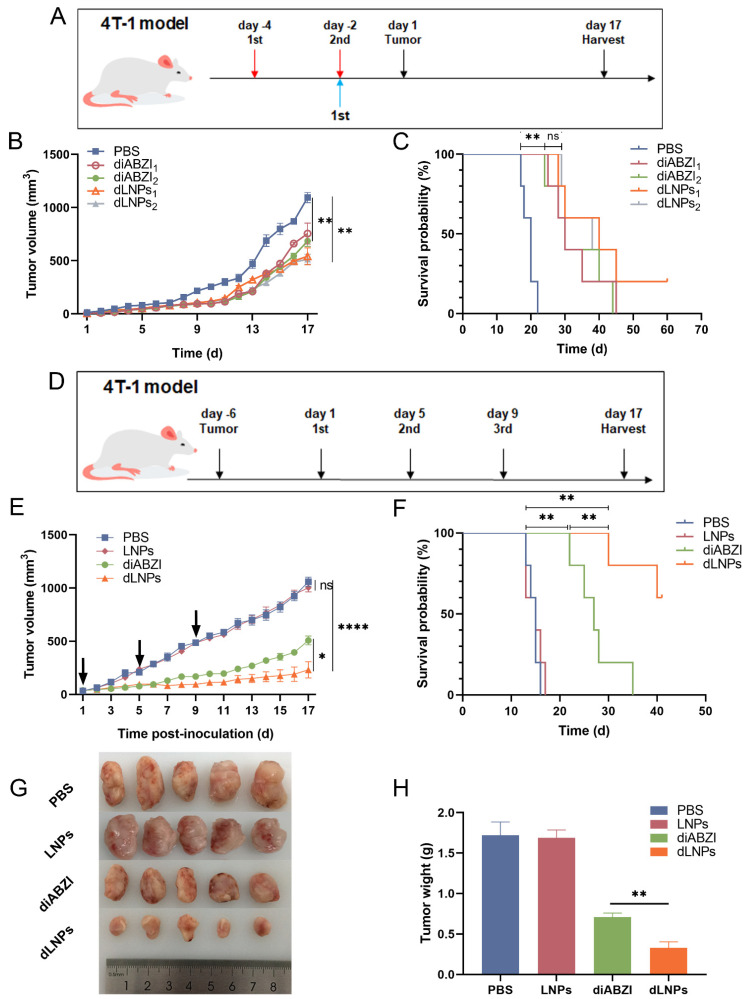
The preventive effect and tumor growth inhibition of dLNPs in vivo (*n* = 5). (**A**) The mice in the diABZI_1_ and dLNPs_1_ groups were treated once on day −2. The mice in the diABZI_2_ and dLNPs_2_ groups were treated twice on days −4 and −2. (**B**) 4T-1 tumor volume in the PBS, diABZI_1_, diABZI_2_, dLNPs_1_, and dLNPs_2_ groups. (**C**) The survival probability of BALB/c mice in the PBS, diABZI_1_, diABZI_2_, dLNPs_1_, and dLNPs_2_ groups. (**D**) The mice were treated three times on days 1, 5, and 9 (*n* = 5). (**E**) The tumor volume in the mice after administration. (**F**) Survival probability of remaining mice over 40 days. (**G**) Photographs of tumors harvested on day 17. (**H**) Average tumor weights of (**G**). ** *p* < 0.01.

The tumor volume of the mice treated with diABZI and dLNPs was smaller compared with the PBS group (Figure 3B), which indicated that the innate immune response generated by the STING pathway activated by the diABZI suppressed tumor growth to a certain extent. Additionally, the survival time of the mice was prolonged after treatment with diABZI_1_, dLNPs_1_, diABZI_2_, and dLNPs_2_ (Figure 3C). The results summarized above demonstrate that the diABZI and dLNPs exhibited a preventive effect on 4T-1 breast cancer.

### 2.6. The Tumor Growth Inhibition of dLNPs

When the tumor volume in the mice reached 100 mm^3^, the mice were treated with PBS, LNPs, diABZI, and dLNPs. There was no difference in tumor volume between the LNPs group and the PBS group mice, indicating that LNPs had no anti-tumor effect. The tumor volume in the diABZI and dLNPs groups was smaller than it was in the PBS group, indicating that diABZI and dLNPs can inhibit tumor growth (Figure 3E). Notably, the tumor volume in the mice treated with the dLNPs was the smallest, suggesting that the dLNPs exhibited a higher tumor inhibition effect than the diABZI in the 4T-1 breast cancer model. The weight and volume of the tumors in the mice correspond to each other (Figure 3G,H), further proving the above results.

The survival probabilities of the different groups were recorded for 40 days. On day 15, all the mice in the PBS group died, while the mice in the diABZI group were all dead on day 35. However, 60% of the mice in the dLNPs group were still alive on day 40 (Figure 3F). Our results show that dLNPs could prolong the survival time of mice in the 4T-1 tumor model.

### 2.7. The Expression of IFN-β

**Figure 4:**
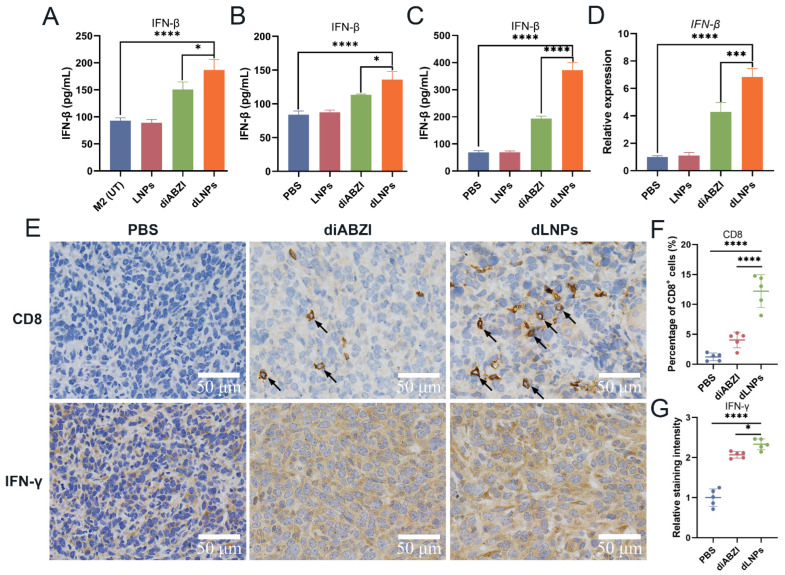
Immunohistochemistry and IFN-β expression. (**A**) The expression of IFN-β in M2-type macrophages treated with LNPs, diABZI, and dLNPs. The expression of IFN-β in untreated M2-type macrophages as a control. (**B**) The expression of IFN-β in the plasma of tumor-bearing mice treated with PBS, LNPs, diABZI, and dLNPs, respectively. (**C**) The expression of IFN-β in tumors of mice treated with PBS, LNPs, diABZI, and dLNPs. (**D**) The relative expression of IFN-β mRNA in tumors of mice treated with PBS, LNPs, diABZI, and dLNPs. (**E**) Immunohistochemistry of mouse tumor tissues; positive cells are stained brown. Scale bar: 50 μm. (**F**) Percentage of CD8^+^ cells in tumor tissues. (**G**) Relative staining intensity of IFN-γ in tumor tissues. * *p* < 0.05, *** *p* < 0.001, **** *p* < 0.0001.

When the STING pathway was activated by the dLNPs, type Ⅰ IFN was released. The expression of IFN-β was assayed to evaluate the levels of the type Ⅰ IFN. The expression of the IFN-β was higher in the M2-type macrophages treated with the dLNPs than in those treated with diABZI (Figure 4A). The expression of IFN-β in the plasma of the tumor-bearing mice treated with dLNPs was also higher than it was in the mice treated with the diABZI (Figure 4B). The expression level of IFN-β and the relative expression level of IFN-β mRNA in the tumors of mice treated with dLNPs three times were analyzed. The results showed that the expression of IFN-β and the expression level of IFN-β mRNA in the tumor tissues of the mice treated with dLNPs were significantly higher than those in the diABZI group, LNPs, and PBS groups (Figure 4C,D). Our results suggest that dLNPs can enter tumor cells and macrophages through the cell membrane and release diABZI in the cytoplasm. The diABZI binds to the STING proteins on the endoplasmic reticulum and induces the expression of type I IFN.

### 2.8. Immunohistochemical of Tumor Tissue

Immunohistochemical analysis was performed on the tumor tissues of the mice after the antitumor treatment. Compared with the PBS group, the cytotoxic CD8^+^ T cell population was increased after treatment with diABZI or dLNPs. Furthermore, the number of cytotoxic CD8^+^ T cells in the tumors in the dLNPs group was greater than that observed in the diABZI group (Figure 4E). It has been shown in several studies that type I IFN can induce CD8^+^ T cell activation to exert antitumor effects [25,26]. The dLNPs exhibited higher cytotoxic CD8^+^ T cell recruitment efficiency (12.2%) than the PBS and LNPs (Figure 4F). Our results showed a significant increase in IFN-γ expression in the tumors in the diABZI and dLNPs groups (Figure 4E,G) due to the increased number of CD8^+^ T cells in the tumor microenvironment being able to generate more IFN-γ [27]. 

### 2.9. Toxicity Analysis of dLNPs In Vitro

**Figure 5:**
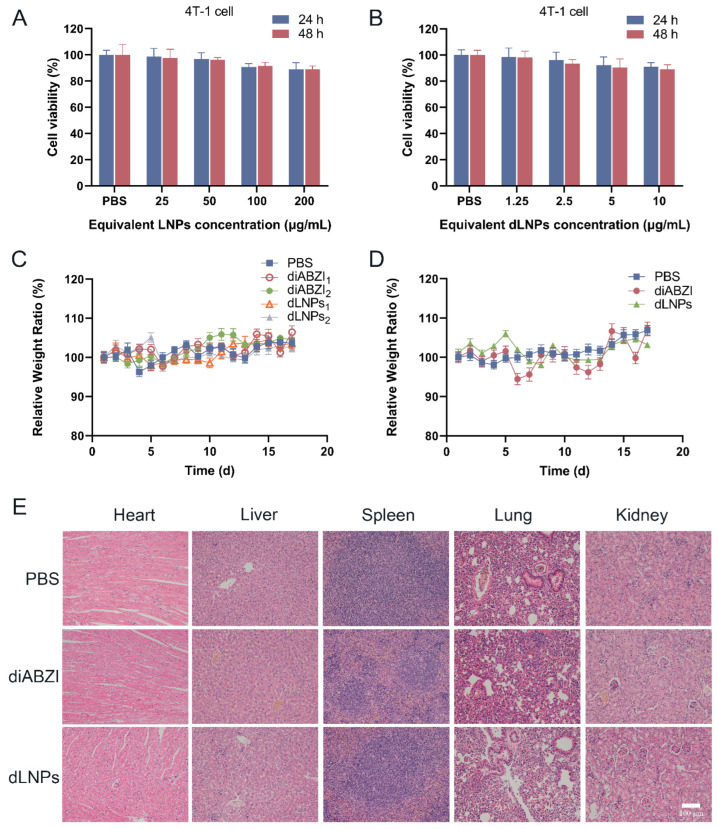
Safety evaluation of dLNPs in vitro and in vivo. (**A**) The viability of 4T-1 cells treated with PBS and 25, 50, 100, or 200 μg/mL LNPs for 24 h and 48 h. (**B**)The viability of 4T-1 cells treated with PBS and diABZI concentrations (1.25, 2.5, 5, or 10 μg/mL) of dLNPs for 24 h and 48 h. (**C**) The bodyweight curves of different groups of mice in the prevention and (**D**) treatment experiments. (**E**) H&E staining of organs harvested on day 17.

To evaluate the safety of the LNPs and dLNPs, the cell viability of the 4T-1cells was measured by a CCK-8 assay after co-culturing with different concentrations of dLNPs for 24 h and 48 h. Compared with the PBS and LNPs groups, the viability of the 4T-1 cells was not affected by the LNPs at various concentrations after being left to co-culture for 24 h or 48 h (Figure 5A). Briefly, the LNPs exhibited negligible toxicity to 4T-1 cells, even when the concentration reached 200 µg/mL. Similarly, there was no significant decrease in cell viability when the concentration of the dLNPs was in the range of 1.25–10 ng/µL (Figure 5B). This result indicates that the LNPs and dLNPs had no direct killing effect on the 4T-1 cells. This result also demonstrates that the dLNPs exert antitumor effects through the activation of STING-mediated innate immunity in vivo.

Hemolysis tests showed that dLNPs (10 ng/µL) have a hemolysis rate of 2.82% (Figure S1), which is lower than 5%, proving their high biosafety. They can also be administered by intravenous injection.

### 2.10. Safety Evaluation In Vivo

To monitor the toxicity of dLNPs, the bodyweight changes in the mice were precisely recorded every day after administration. In the experiments to determine the prevention and antitumor effects, there was no significant bodyweight loss in the diABZI group or dLNPs group (Figure 5C,D), demonstrating that the dLNPs possess excellent biosafety. After 17 days, the major organs of the mice, including the heart, liver, spleen, lung, and kidney, were harvested for H&E staining. The organs taken from the mice in the PBS, diABZI, and dLNPs groups had no obvious damage (Figure 5D), which demonstrates that the dLNPs possess high security.

## 3. Materials and Methods

### 3.1. Materials

Hydrogenated soybean phosphatidylcholine (HSPC), 1,2-distearoyl-sn-glycero-3-phosphoethanolamine-N-[methoxy (polyethylene glycol)-2000] (DSPE-PEG2000), and cholesterol were purchased from Xi’an ruixi Biological Technology Co., Ltd. (Xi’an, China). Indocyanine green (ICG) for injection was purchased from Dandong yichuang Pharmaceutical Co., Ltd. (Dandong, China).

### 3.2. Preparation of LNPs, dLNPs, and ICG-LNPs

HSPC, cholesterol, and DSPE-PEG2000 were dissolved in an anhydrous ethanol solution (Aladdin, Shanghai, China) at the ratio of 56.3:38.4:5.3 (molar ratio). The LNPs (10 mg/mL) were prepared by ethanol injection combined with polycarbonate film extrusion.

ICG-LNPs were prepared by the same method using ICG with a concentration of 2 mg/mL as the aqueous phase for the liposomes. 

The liposomes for drug delivery were prepared using the ethanol injection method with 250 mM of ammonium sulfate as the internal water phase, and the external water phase was transformed into a phosphate-buffered solution (PB, pH 7.4) via a dialysis bag (300 KD). The preparation process for the dLNPs can be described as follows: 0.03 mg of diABZI was dissolved in 3 μL of DMSO (Sigma-Aldrich, Saint Louis, MO, USA); then, 100 μL of LNPs was added into the above solution and heated in a water bath (65 °C, 30 min). The diABZI was encapsulated into liposomes using the ammonium sulfate gradient method. The dLNPs were obtained after dialysis to remove the free diABZI that had not been encapsulated into the liposomes.

### 3.3. Liposome Characterization

The particle size of the liposomes was measured by dynamic light scattering (DLS) using a Malvern Zetasizer Nano ZSE (Malvern, UK). The zeta potential was determined on the same machine using the electrophoretic light scattering (ELS) technique. In addition, the morphology of the liposomes was measured using a transmission electron microscope (TEM, Joel, Tokyo, Japan). The encapsulation efficiency (*EE*%) was measured using high-performance liquid chromatography (HPLC). The encapsulation efficiency and drug-loading capacity were calculated using the following formulas:

(1) $$EE=\frac{diABZI\;losded\;in\;liposome}{\mathrm{innitial}\;\mathrm{amounts}\;\mathrm{of}\;\mathrm{diABZI}}\times 100\%$$

(2) Drug loading=dosage×EE

### 3.4. Cell Culture

Mouse breast cancer cells (4T-1) were purchased from the cell bank of the Chinese Academy of Sciences (Shanghai, China). These cells were cultured in RPMI-1640 (Thermo Fisher Scientific, New York, NY, USA ) with 10% fetal bovine serum (FBS) (Thermo Fisher Scientific, New York, NY, USA) at 37 °C and 5% CO_2_.

Mouse bone marrow-derived macrophages (BMDMs) were isolated from 8-week-old BALB/c mice and cultured in DMEM medium supplemented with 10% FBS, 1% penicillin/streptomycin (Hyclone, Logan, UT, USA), and 20 ng/mL macrophage colony-stimulating factor (M-CSF, PeproTech, Cranbury, NJ, USA). Macrophages were obtained after 6 days. After 24 h of interleukin-4 (IL-4, 25 ng/mL, PeproTech, Cranbury, NJ, USA) induction, the macrophages were induced into M2-type macrophages.

### 3.5. Cytotoxicity Analysis of dLNPs In Vitro

For the cytotoxicity analysis, 4T-1 cells were seeded in 96-well culture plates at 5 × 10^3^/well and incubated at 37 °C in 5% CO_2_ for 24 h. Then, the LNPs were added to each well to make final LNPs concentrations of 25, 50, 100, and 200 μg/mL. dLNPs were added to each well to make final diABZI concentrations of 1.25, 2.5, 5, and 10 μg/mL, respectively. The 4T-1 cells were cultured at 37 °C with 5% CO_2_ for another 24 h. Then, 10 µL of Cell Counting Kit-8 (CCK-8, Merck KGaA, Darmstadt, Germany) was added to each well and cultured for 4 h, and the optical density (O.D.) was determined to be at a wavelength of 450 nm using a microplate reader (Biotek, Winooski, VT, USA). The experiment was repeated three times. Finally, the cell viability was calculated using the following formula:

(3) $$Cell\;viability=\frac{As-Ab}{Ac-Ab}\times 100\%$$

where As, Ac, and Ab represent the absorbance of the sample wells, control wells, and blank wells, respectively.

The hemolysis assay was used to evaluate the biocompatibility of the dLNPs. We took 2 mL of mouse blood and centrifuged it at 4 °C (1000× *g* rpm) to remove the supernatant. The RBCs were washed with PBS three times until the supernatant was colorless. Then, 10 mL of normal saline was added to resuspend the erythrocytes. Next, 900 µL of dLNPs was added to 100 μL of erythrocyte suspension to make the final concentration of 10 μg/mL. The mixture was incubated in a water bath at 37 °C for 1 h. Then, it was centrifuged at 4 °C (1000× *g* rpm) for 10 min, during which time we observed hemolysis, and a microplate reader was used to detect the OD value of the supernatant at a wavelength of 541 nm. The PBS group was used as the negative control, and the 2% triton X-100 group was used as the active control. The hemolysis rate was calculated according to the following formula:

(4) $$\mathrm{Hemolysis}\;\mathrm{rate}=\frac{{OD}_{EG}-{OD}_{NC}}{{OD}_{AC}-{OD}_{NC}}\times 100\%$$

where *EG*, *NC*, and *AC* represent the experimental group, negative control, and active control, respectively.

### 3.6. In Vitro Drug Release Study

diABZI (0.03 mg/100μL) and dLNPs (0.03 mg/100 μL) were added into dialysis bags (300 KD), and then placed in 5 mL of PBS (pH = 7.4) solution for incubation at 37 °C, respectively. Then, 100 μL of the external solution was taken at 10 min, 30 min, 1 h, 2 h, 4 h, 8 h, 16 h, 24 h, and 48 h. An equal volume of PBS was added after each sampling period. For the mobile phase, a 90% acetonitrile solution (Aladdin, Shanghai, China) was used, and the flow rate was 1 mL/min. The absorbance of the diABZI in the external dialysate at 256 nm was determined by HPLC for quantitative analysis.

### 3.7. Pharmacokinetic Study

First, diABZI (0.03 mg/per mouse) and diABZI concentrations (0.03 mg/per mouse) of the dLNPs were injected intravenously into BALB/c mice (6 weeks, female, 20–25 g). Then, 100 μL of eyeball blood was collected at 10 min, 30 min, 1 h, 2 h, 4 h, 8 h, 12 h, 24 h, 36 h, and 48 h after administration and placed into 1.5 mL centrifuge tubes with 10 μL of sodium citrate (30 mg/mL). After centrifugation in a high-speed freezing centrifuge (Eppendorf, Germany) at 4 °C (3000× *g* rpm) for 4 min, plasma was obtained. Then, the plasma and acetonitrile were mixed in a volume ratio of 1:1 and vortexed for 10 s. The diABZI in the sample was measured by HPLC (Thermo Fisher Scientific, New York, NY, USA).

### 3.8. In Vivo Passive Targeting Efficiency of LNPs

When the tumor size in the 4T-1 model mice was 200 mm^3^, 100 μL of free ICG (2 mg/mL) and 100 μL of ICG-LNPs (2 mg/mL) were injected intravenously. Intravital imaging was performed at 0.5 h, 1 h, 2 h, 4 h, 8 h, 16 h, 24 h, and 48 h to observe the fluorescence intensity of ICG in the tumors. At 48 h, the mice were killed, and the tumor, heart, liver, spleen, lung, and kidney were harvested for fluorescence imaging. Their fluorescence intensity was quantified.

### 3.9. In Vivo Evaluation of the Preventive Effect

BALB/c mice (6 weeks, female) were randomly divided into five groups: diABZI_1_, diABZI_2_, dLNPs_1_, dLNPs_2_, and PBS. The diABZI_1_ and dLNPs_1_ groups were treated once with diABZI (0.03 mg/per mouse) and diABZI concentrations (0.03 mg/per mouse) of dLNPs, respectively. Additionally, the diABZI_2_ and dLNPs_2_ groups were treated twice with diABZI (0.03 mg/per mouse) and diABZI concentrations (0.03 mg/per mouse) of dLNPs, respectively. The PBS group was injected with PBS as a control. On day 1, 0.1 mL of RPMI-1640 with 1 × 10^6^ 4T-1 cells was subcutaneously injected into the right flank of each mouse. In addition, the body weight and tumor size were recorded daily until day 17. The *Tumor volume* was calculated using the formula:

(5) $$Tumor\;volume=\frac{length\times {width}^{2}}{2}$$

The survival rate was recorded for 60 days. The mice were humanely euthanized when the tumor ulcerated, the tumor volume reached 1500 mm^3^, or the bodyweight decreased by 20%.

### 3.10. In Vivo Evaluation of the Antitumor Effect

The 4T-1 tumor models were prepared by subcutaneously injecting 0.1 mL of 4T-1 cells at a concentration of 1 × 10^6^/mL into the right flank of each mouse (6 weeks, female). The mice were randomly divided into four groups, with five mice in each group. The day when the tumor volume reached 100 mm^3^ was considered day 1. Starting at day 1, diABZI concentrations (0.03 mg/per mouse) of dLNPs were delivered by intravenous injection every four days for a total of three administrations. At the same time, PBS, LNPs, and diABZI were delivered as controls. The bodyweight, tumor size, and survival rate were recorded. On day 17, the tumor, heart, liver, spleen, lung, and kidney were harvested. The expression of CD8 and IFN-γ in the tumor tissues was analyzed by immunohistochemistry. The main organs were stained with H&E to analyze the safety of the dLNPs in vivo.

### 3.11. Expression Analysis of IFN-β

IFN-β is a type of secreted protein. After stimulation with dLNPs for 24 h, the IFN-β expression in the culture medium of M2-type macrophages was measured using a mouse IFN-β ELISA kit (Mlbio, Shanghai, China).

At 6 h after being treated with PBS, LNPs, diABZI, and dLNPs, plasma from the tumor-bearing mice was harvested for IFN-β expression assays.

The expression level of IFN-β in the mouse tumors that were treated with dLNPs was investigated. Specifically, after administering PBS, LNPs, diABZI, and dLNPs to the mice three times, the tumors were harvested. An amount of 0.5 g of tumor tissue was homogenized in 1 mL pre-cooled NaCl solution (0.9%). The tissue homogenate was centrifuged, and the expression level of the IFN-β was measured using a mouse IFN-β ELISA kit (Mlbio, Shanghai, China). Similarly, total RNA from mouse tumor tissues was extracted and used to detect the expression level of IFN-β mRNA.

### 3.12. Immunohistochemistry

The mouse tumors were embedded in paraffin, and the tissues were cut into 5 μm-thick sections. Then, the paraffin was removed, and the tissue was rehydrated. The sections were immersed in antigen retrieval solution and then microwaved and boiled for 15 min before being left to cool to room temperature in the antigen retrieval solution. After the sections were washed with PBS, 3% BSA was added and blocked for 30 min at room temperature. The sections were stained with CD8 and IFN-γ antibodies overnight at 4 °C. After washing with PBS, HRP-labeled secondary antibody was added and incubated at room temperature for 1 h. The nuclei were stained with DAPI for 30 min.

### 3.13. Ethical Declarations

All the animal experiments complied with the ARRIVE guidelines and were carried out in accordance with the National Institutes of Health Guide for the Care and Use of Laboratory Animals and the guidelines approved by the Special Committee on Scientific Research Ethics of Liaocheng University (Approval Code: 2022111010; Approval Date: 1 November 2022).

### 3.14. Statistical Analysis

All the statistical analyses were performed using GraphPad Prism 8.0.2. Statistical significance was assayed by a t-test or one-way ANOVA test. Moreover, the log-rank (Mantel–Cox) test was used for the survival analyses. All the data were expressed as the mean ± standard deviation. The *p*-values less than 0.05 were considered statistically significant.

## 4. Discussion

Liposomes are frequently used for the delivery of antitumor drugs because of their biosafety and biocompatibility [28,29,30]. The particle size, uniformity, and drug release rate of liposomes are important factors for liposome evaluation. Negatively charged liposomes also exhibit enhanced cellular uptake efficiency [31,32]. In this study, we developed a liposomal formulation encapsulating diABZI (dLNPs) to improve its antitumor efficacy against breast cancer. The dLNPs demonstrated favorable diABZI encapsulation efficiency, as well as high uniformity and stability.

The drug release of the liposomal formulations that are administered intravenously into the bloodstream is an important factor that must be considered [28]. In this study, the drug release rate of the dLNPs was evaluated by simulating the blood environment in vitro. At the same time points, the concentration of diABZI in the dialysis external solution of the dLNPs was lower than that in the free diABZI solution. Therefore, the results indicate that dLNPs exhibit a slow-release effect. To examine the in vivo metabolic rate of dLNPs, pharmacokinetic experiments were conducted [33]. Compared with the free diABZI, the dLNPs exhibited a slower clearance rate in the bloodstream of the mice and an increased circulating capacity in the blood, resulting in the accumulation of the dLNPs in the tumor tissues. Previous studies have demonstrated that intravenously injected liposomes are capable of passively targeting tumor tissue via the EPR effect [34]. Live fluorescence imaging was conducted on mice that were injected with free ICG and ICG-LNPs, and it was observed that the ICG-LNPs exhibited a slower metabolic rate compared to free ICG. This finding is consistent with the results of pharmacokinetic experiments. Additionally, the prolonged in vivo circulation time of ICG-LNPs facilitated the accumulation of these nanoparticles in tumor tissues due to the EPR effect.

In the prevention experiment, it was shown that both diABZI and dLNPs had a preventive effect on 4T-1 breast cancer cells. However, the experimental findings revealed no significant differences among the diABZI1, dLNPs1, diABZI2, and dLNPs2 groups. This could be due to the fact that innate immunity also possesses immunological memory, although its specificity is lower and its duration shorter than that of adaptive immunity [35]. Subsequently, the 4T1 tumor-bearing mice were treated with PBS, diABZI, and dLNPs. The results indicated that the dLNPs exhibited a greater tumor inhibition effect than the diABZI in the 4T-1 breast cancer model. Additionally, the dLNPs prolonged the survival time of the studied mice.

Several studies have demonstrated that type I IFN can activate CD8^+^ T cells, which, in turn, can exert antitumor effects [25,26]. The dLNPs demonstrated high efficiency in recruiting cytotoxic CD8^+^ T cells. Our results revealed a significant increase in IFN-γ expression in the tumors of both the diABZI and dLNPs groups. This is likely due to the increased presence of CD8^+^ T cells in the tumor microenvironment, which can generate more IFN-γ.

## 5. Conclusions

In this study, dLNPs exhibited high encapsulation efficiency and demonstrated the ability to passively target tumor tissues and extend the duration of action in vivo. Furthermore, dLNPs are highly effective at enhancing the expression of IFN-β and IFN-γ, as well as at recruiting CD8^+^ T cells to concentrate in tumor tissues, thereby remodeling the tumor microenvironment. Compared to the free diABZI, the dLNPs demonstrated stronger tumor growth inhibition and prolonged survival in the 4T-1 model mice. Therefore, the use of liposomes to improve the poor serological stability of diABZI is a feasible strategy.

## Figures and Tables

**Table 1:** Size and zeta potential of LNPs and dLNPs.

Sample	Z-Average ^a^ (nm)	PDI ^b^	Zeta Potential (mV)	EE ^c^ (%)
LNPs	81.14 ± 0.586	0.209 ± 0.0196	−8.96 ± 0.466	-
dLNPs	99.76 ± 0.230	0.225 ± 0.0076	−6.74 ± 0.896	58.29 ± 0.53

^a^ The values are means ± standard deviations (*n* = 3); ^b^ polydispersity index; ^c^ encapsulation efficiency.

## Data Availability

Not applicable.

## Supplementary Materials

The following supporting information can be downloaded at: https://www.mdpi.com/article/10.3390/molecules28093740/s1.

## Sample Availability

The data that support the findings of this study are available from the corresponding author upon reasonable request.
