# Supplementary material for: Anticancer Effect of STING Agonist-Encapsulated Liposomes on Breast Cancer
Source: Molecules. 2023 Apr 26;28(9):3740. doi: 10.3390/molecules28093740 (PMC10179927; doi:10.3390/molecules28093740)
Supplement: Supplementary file 1 [file molecules-28-03740-s001.zip › molecules-2331896-supplementary.pdf]

Supplementary Material

Preparation of LNPs, dLNPs, and ICG-LNPs

The anhydrous ethanol solution was quickly injected into the swirling ammonium sulfate solution (250 mM, 65℃) using a syringe with a 0.33 mm needle and then heated in a water bath at 65℃ with stirring for 30 min. Subsequently, ethanol was removed from the liposomes using a dialysis bag (300 KD), in which ammonium sulfate was used as dialysate. Afterward, the liposomes were repeatedly extruded 21 times using a liposome extruder loaded with polycarbonate membranes of 200 nm, 100 nm, and 50 nm (Avanti, USA) in turn. Eventually, empty liposome nanoparticles (LNPs) were obtained.

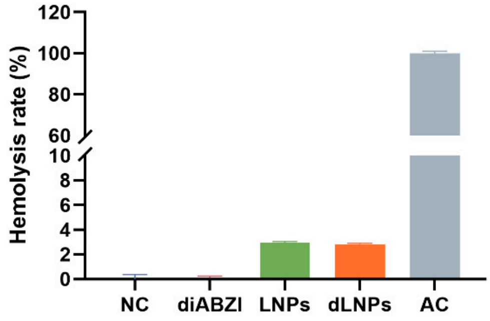

Figure S1. Hemolysis rate of dLNPs

Primers used in this study

Sequences for primers (listed 5′ to 3′)

β-actin F: GGCACCACACCTTCTACAATG

β-actin R: GAGGCATACAGGGACAGCAC

IFN-β F: CCACAGCCCTCTCCATCAACT

IFN-β R: TGGATGGCAAAGGCAGTGTAA
